# Long‐coronavirus disease among people living with HIV in western India: An observational study

**DOI:** 10.1002/iid3.467

**Published:** 2021-06-02

**Authors:** Sanjay Pujari, Sunil Gaikwad, Abhishek Chitalikar, Digamber Dabhade, Kedar Joshi, Vivek Bele

**Affiliations:** ^1^ Institute of Infectious Diseases Pune India

**Keywords:** HIV, India, long‐COVID, prevalence, risk factors

## Abstract

**Background:**

Long‐COVID is emerging as a significant problem among individuals who recovered from COVID‐19. Scant information is available on the prevalence, characteristics, and risk factors for long‐COVID among people living with HIV (PLHIV).

**Setting:**

A tertiary level, private, HIV clinic in western India.

**Methods:**

A prospective, observational study was conducted to assess the prevalence of long‐COVID among PLHIV. Long‐COVID was defined as the presence of at least one symptom after 30 days of illness onset. A questionnaire for assessing general, cardiorespiratory, neuro‐psychiatric, and gastro‐intestinal symptoms was used to screen individuals with history of confirmed COVID‐19. Data on demographics, HIV‐related variables, comorbidities, and severity of COVID‐19 were abstracted from electronic medical records. Univariate and multivariate logistic regression were used to identify risk factors for long‐COVID.

**Results:**

Ninety‐four PLHIV were screened for long‐COVID. Median (interquartile range [IQR]) age was 51 (47–56) years and 73.4% were males. The majority (76.6%) had a history of asymptomatic–mild COVID‐19 illness. The prevalence of long‐COVID was 43.6% (95% confidence interval [CI], 33.4–54.2). Moderate–severe COVID‐19 illness was significantly associated with long‐COVID (adjusted odds ratio, 4.7; 95% CI, 1.4–17.9; *p* = .016). Among individuals with long‐COVID, cough (22.3%) and fatigue (19.1%) were the commonest symptoms. The median (IQR) duration for resolution of symptoms was 15 (7–30) days. Ten individuals (10.6%) had persistent symptoms at a median of 109 days since the onset of COVID‐19.

**Conclusion:**

Long‐COVID is common among PLHIV with moderate–severe acute COVID‐19 illness. There is a need for integration of long‐COVID diagnosis and care services within antiretroviral therapy clinics for PLHIV with COVID‐19.

## Graphical abstract

Long‐COVID‐19 was documented among 43.6% of people living with HIV in western India. Moderate/severe COVID‐19 illness was independently associated with long COVID‐19 in this cohort.

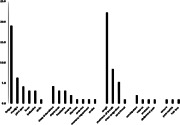

## 1 INTRODUCTION

Coronavirus disease 19 (COVID‐19) has emerged into a global pandemic of an unprecedented emergency. Acute infection due to severe acute respiratory coronavirus‐2 (SARS‐COV‐2) has been the focus of pathophysiology and clinical management research as it is associated with high morbidity and mortality. However, the natural history of SARS‐COV‐2 infection has been characterized with at least two late clinical presentations in some individuals: a post‐acute hyperinflammatory illness and late sequelae (also labeled as long‐COVID or long‐haulers).^1^ The definition of long‐COVID is not standardized although the persistence of, or occurrence of new symptoms or laboratory/radiological abnormalities (cardiovascular, pulmonary, and neuropsychiatric) after approximately 4 weeks of initial infection has been used by studies.

In a meta‐analysis of studies on long‐COVID, 80% (95% confidence interval [CI], 65–92) of individuals had one or more long‐term symptoms when assessed two or more weeks after initial symptoms.^2^ However, evidence on stratification of long‐COVID symptoms based on age, gender, comorbidities, and severity of acute illness were lacking in all the included studies. The exact pathophysiological mechanisms contributing to long‐COVID are currently unclear. Immune‐mediated mechanisms (protracted inflammation, autoimmunity), persistent viral reservoirs or fragments of viral RNA, and genetic predisposition have been proposed to contribute to long‐COVID and are being studied systematically.^3^ Chronic inflammation, immune dysfunction, and viral persistence are also documented among people living with HIV (PLHIV) on effective antiretroviral therapy (ART).^4^ Whether these overlapping mechanisms further increase the risk of long‐COVID in PLHIV needs further investigation. Moreover, some symptoms in individuals with long‐COVID may overlap with conditions common in chronic human immunodeficiency virus (HIV) infection, especially neuro‐cognitive/psychiatric problems.

While the interaction between HIV and acute SARS‐COV‐2 infection has been well studied, there is a paucity of data on the prevalence of long‐COVID among PLHIV. We designed a study to determine the prevalence and characteristics of long‐COVID among PLHIV and identify associated risk factors.

## 2 METHODS

### 2.1 Design

This study is a prospective, observational study conducted between November 1, 2020 and January 31, 2021.

### 2.2 Setting

A tertiary level, private, urban HIV clinic in western India.

### 2.3 Ethics statement

Individuals provide an Independent Ethics Committee‐approved written informed consent for using routinely collected clinical and laboratory data for research analysis and publication at the time of clinic registration.

### 2.4 Participants

All adult PLHIV (≥18 years) with a confirmed diagnosis of COVID‐19 were eligible. Diagnosis of COVID‐19 was confirmed with reverse‐transcriptase polymerase chain reaction or rapid antigen test. The COVID‐19 clinical illness severity were categorized based on the Ministry of Health and Family Welfare (Government of India) criteria into mild (upper respiratory tract infection without breathlessness or hypoxia), moderate (pneumonia with SpO_2_ <94% but >90%), severe (pneumonia with SpO_2_ <90%), and critical (sepsis, septic shock, and acute life‐threatening organ dysfunction).^5^

### 2.5 Procedures

A standardized questionnaire for assessing long‐COVID symptoms was developed. Symptoms were categorized as general (fever, chills, body aches, fatigue, myalgia, joint pains, sweats, and rash), NP (headache, loss of smell, loss of taste, dizziness, confusion, sleep disturbance, memory impairment, depression, and anxiety), cardiopulmonary (CP) (cough, sore throat, sinus congestion, shortness of breath, chest pain, chest tightness, and wheezing) and GI (nausea, anorexia, vomiting, diarrhea, constipation, and abdominal pain).

The questionnaire was administered telephonically or on follow‐up visits. Individuals were asked about the current presence of or retrospectively recollect the existence of symptoms 30 days after illness onset. Date of onset and duration to symptoms resolution or persistence were documented. For PLHIV with reported symptoms, a repeat telephonic assessment after 4–8 weeks was conducted to assess persistence/resolution of symptoms. Management advice was provided.

Data on demographics, HIV‐related characteristics (closest CD4 count, HIV viral load to date of assessment), medical comorbidities, and acute COVID‐19 (illness category, treatments, duration of illness) were abstracted from electronic medical records.

### 2.6 Statistical analysis

Long‐COVID was defined as the presence of at least one symptom after 30 days of illness onset. Data were summarized by the presence or absence of long‐COVID using median and interquartile range (IQR) for continuous variables and frequencies and percentages for categorical variables. Continuous and categorical variables were compared using Student's *t* test, or Fischer's exact test as appropriate. Univariate and multivariate logistic regression were performed to identify risk factors associated with long‐COVID. *p* < .05 were considered to be statistically significant.

The rate of occurrence of specific long‐COVID symptoms was summarized using frequencies and percentages. All analyses were performed, and graphs created using Prism 9 (version 9.0.1; GraphPad Software, LLC).

## 3 RESULTS

One‐hundred and ten PLHIV with a confirmed COVID‐19 diagnosis were screened for eligibility. Nine individuals had died owing to acute COVID‐19 illness, four could not be contacted, and three individuals had not completed 30 days since illness onset. Ninety‐four PLHIV were screened for long‐COVID and included in the final analysis. The screening was done telephonically for 61 (64.9%) individuals.

### 3.1 Baseline characteristics

The median (IQR) age of included individuals was 51 (47–56) years and 73.4% were males (Table 1). Median (IQR) duration since HIV diagnosis was 15 (12–18) years and the proportion of individuals with CD4 count <200/mm^3^ and PVL < 200 copies/ml closest to assessment were 6.4% and 91.5%, respectively. All individuals were on ART with 47.9% and 40.4% on integrase strand transfer inhibitor (INSTI) and non‐nucleoside reverse‐transcriptase inhibitor‐based regimens, respectively. At least one medical comorbidity was present among 46.8% of individuals. The commonest comorbidities included hypertension (39.4%) and diabetes (17.0%).

**Table 1:** Clinical characteristics and risk factors for long‐COVID among people living with HIV

Characteristics	Overall *n* (%)	Long‐COVID		Univariate analysis		Multivariate analysis	
		Yes (*n* = 41)	No (*n* = 53)	OR (95% CI)	*p* value	OR (95% CI)	*p* value
Gender							
Female	69 (73.4)	32 (78.0)	37 (69.8)	Ref	–	Ref	–
Male	25 (26.6)	9 (22.0)	16 (30.2)	1.5 (0.6–3.7)	.481	1.9 (0.6–6.3)	.232
Age (years) >50	48 (51.1)	24 (58.5)	24 (45.3)	1.7 (0.8–3.9)	.219	1.0 (0.9–1.1)	.262
Duration of HIV diagnosis ≥15 years	53 (56.4)	24 (58.5)	29 (54.7)	1.2 (0.5–2.6)	.834	1.1 (0.5–3.0)	.731
CD4 < 200	6 (6.4)	4 (9.8)	2 (3.8)	2.8 (0.6–15)	.398	0.7 (0.1–8.2)	.788
HIV VL > 200 (within 12 months of screening)	3 (3.2)	2 (4.9)	1 (1.9)	0.4 (0.1–3.3)	.578	0.1 (0.1–2.3)	.186
Type of ART							
INSTI	45 (47.9)	22 (53.7)	23 (43.4)	Ref	–	Not included	
NNRTI	38 (40.4)	15 (36.6)	23 (43.4)	1.5 (0.6–3.4)	.506		
PI/r	11 (11.7)	4 (9.7)	7 (13.2)	1.7 (0.4–5.6)	.517		
Presence of any comorbidity	44 (46.8)	23 (56.1)	21 (39.6)	1.9 (0.87–4.5)	.1455	1.6 (0.6‐4.1)	.301
COVID‐19 severity							
Asymptomatic–mild	72 (76.6)	24 (58.5)	48 (90.6)	Ref	.0004*	Ref	.016*
Moderate–severe	22 (23.4)	17 (41.5)	5 (9.4)	6.8 (2.3–18.0)		4.7 (1.4‐17.9)	
Receipt of steroids	18 (19.1)	13 (31.7)	5 (9.4)	4.5 (1.4–12.0)	.008*	Not included	

Abbreviations: ART, antiretroviral; COVID, coronavirus disease; HIV, human immunodeficiency virus; INSTI, integrase‐strand transfer inhibitors; NNRTI, non‐nucleoside reverse‐transcriptase inhibitors; OR, odds ratio; PI/r, protease inhibitor/low‐dose ritonavir; Ref, reference; VL, viral load.

* Significant *p* value.

Acute COVID‐19 illness severity was categorized as asymptomatic, mild, moderate, and severe among 17 (18.1%), 55 (58.5%), 14 (14.9%), and 8 (8.5%), respectively. Twenty‐three individuals (24.5%) received any form of oxygen therapy during acute illness and 19.1% received short‐term steroids.

### 3.2 Prevalence and risk factors for long‐COVID

The prevalence of long‐COVID was 43.6% (95% CI, 33.4–54.2). Characteristics of PLHIV with and without long‐COVID are summarized in Table 1. The prevalence of long‐COVID among asymptomatic, mild, moderate, and severe acute COVID‐19 were 35.3%, 34.6%, 57.1%, and 100%, respectively. On univariate analysis, history of moderate–severe COVID‐19 illness was significantly associated with higher odds of long‐COVID (odds ratio [OR], 6.8; 95% CI, 2.3–18.0; *p* = .0004). Additionally, median CD4 counts closest to screening (*p* = .009) and receipt of steroids (OR, 4.5; 95% CI, 1.4–12.0; *p* = .008) during acute illness were significantly associated with long‐COVID. In logistic regression analysis, only moderate/severe COVID‐19 illness was significantly associated with long‐COVID (adjusted odds ratio, 4.7; 95% CI, 1.4–17.9; *p* = .016).

### 3.3 Long‐COVID symptoms

The median (IQR) duration for the onset of any symptoms was 22 (0–30) days from illness onset while the median (IQR) duration for resolution of symptoms was 15 (7–30) days. Most individuals (68.3%) developed new symptoms post discharge. Ten individuals (10.6%) had ongoing symptoms at last assessment (asymptomatic: 5.9%, mild: 7.2%, moderate: 7.1%, and severe: 37.5%). The median (IQR) duration for persistent symptoms among these individuals was 109 (102–180) days.

**Figure 1:**
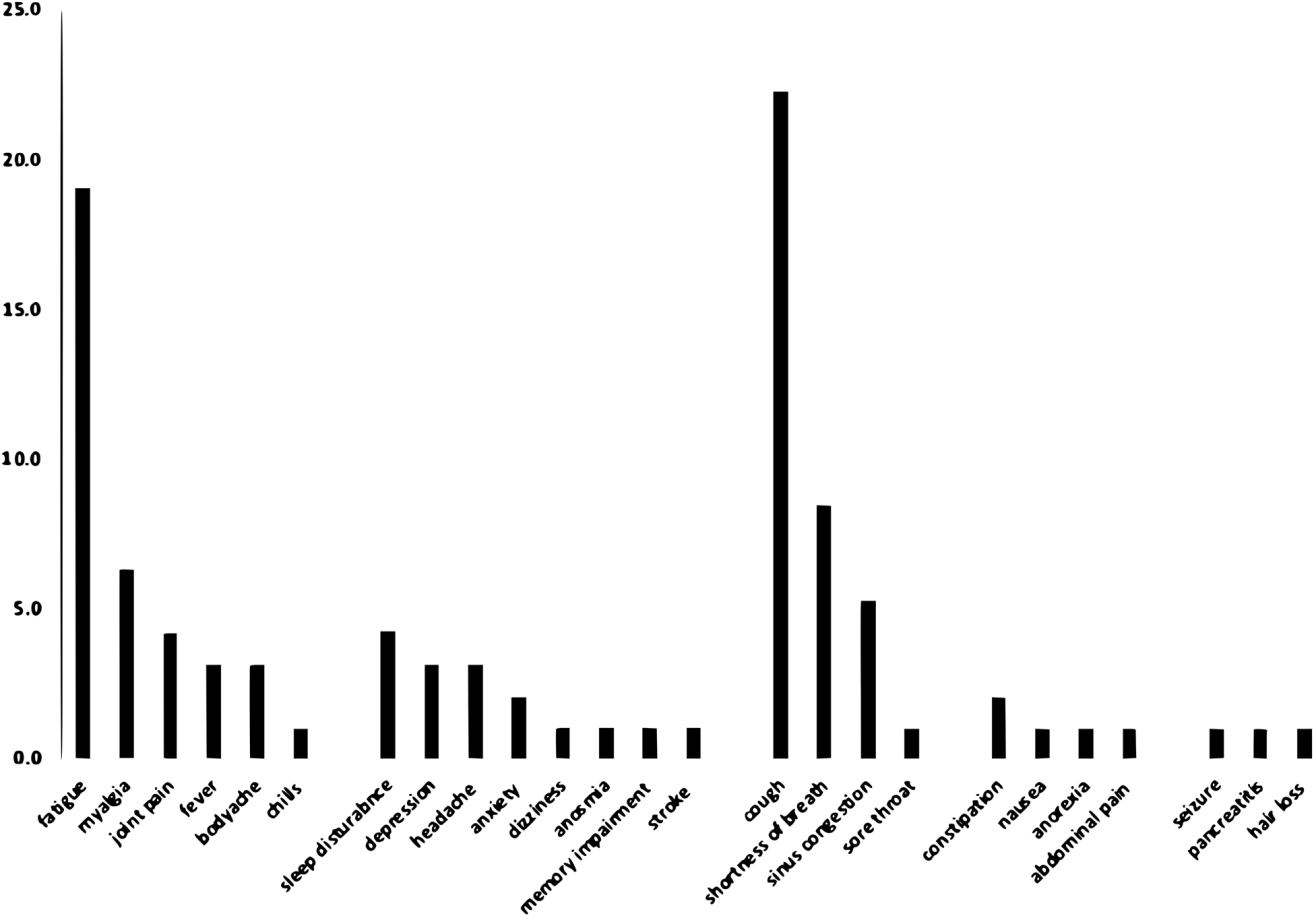
Frequency of symptoms among people living with HIV and long‐COVID

A total of 91 symptoms (general: 35, NP: 16, CP: 35, and GI: 5) was documented among 41 individuals with long‐COVID. Thirty‐two individuals (34.1%) complained of more than one symptom. General with CP symptoms was the commonest combination (*n* = 12). The frequency of various symptoms among long‐COVID is shown in Figure 1. Cough (22.3%) and fatigue (19.1%) were the commonest symptoms followed by shortness of breath (8.5%), myalgia (6.4%), sinus congestion (5.3%), and sleep disturbance (4.3%). All other symptoms were documented in a frequency of <5%.

All individuals with persistent symptoms had at least two categories involved. The frequency of general, NP, CP, and GI symptoms among them were 60.0%, 50.0%, 40.0%, and 20.0%, respectively. The commonest symptoms were fatigue (40.0%), cough (40.0%), shortness of breath (30.0%), and feeling depressed (20.0%). Seizure disorder, acute kidney injury, and acute pancreatitis was diagnosed or self‐reported in one individual each.

## 4 DISCUSSION

We document a prevalence of 43.6% of long‐COVID among PLHIV in western India. However, only 10.6% of individuals had persistent clinical symptoms at a median duration of 109 days from the recovery of acute COVID‐19. At the time of writing, we did not find any peer‐reviewed publication on the rates of long‐COVID among PLHIV.

The absence of a standard definition of long‐COVID is a key challenge in characterizing the true frequency and associated risk factors. The National Institute for Health and Care Excellence (NICE) guideline defines long‐COVID as ongoing symptomatic COVID‐19 (signs and symptoms of COVID‐19 from 4 to 12 weeks) or post‐COVID‐19 syndrome (symptoms continuing for more than 12 weeks and not explained by alternative diagnosis).^6^ However, most studies have used a duration of more than 2 weeks of symptom persistence to define long‐COVID. Large variations in the prevalence of long‐term COVID‐19 ranging from 50% to 94% have been reported.^7^, ^8^, ^9^, ^10^ However major limitations of these studies include variability in definitions and focus on specific symptom category. In the largest, well‐designed cohort study from China, 76% of patients reported at least one symptom at 6 months after acute COVID‐19.^11^ No data are available for long‐COVID from India. Although comparison of prevalence of long‐COVID in our study with historical reports is inappropriate, it is likely that PLHIV do not have a higher rate of long‐COVID as compared with the general population.

The pathophysiology of long‐COVID is unclear. Proposed mechanisms include long‐term sequalae due to perturbations of immune and inflammatory responses during acute COVID‐19, the occurrence of new autoimmune phenomenon and long‐term SARS‐COV‐2 persistence.^12^ Chronic immune activation and autoimmune diseases are common among PLHIV.^13^, ^14^ Whether these underlying pathophysiologic mechanisms amplify the occurrence of long‐COVID among PLHIV needs further research.

Acute COVID‐19 illness severity was the only factor associated with long‐COVID among PLHIV in our study. Interestingly, no HIV‐related variables like CD4 count, viral loads, demographics, and the presence of medical comorbidities were associated with long‐COVID. In the non‐HIV population, a more severe acute COVID‐19 illness was associated with a subsequent increased risk of pulmonary diffusion abnormality, fatigue or muscle weakness, and anxiety or depression.^11^

Conflicting information is available on acute COVID‐19 severity and outcomes among PLHIV. Two large studies documented higher rates of severe disease needing hospitalization and mortality due to COVID‐19 among PLHIV.^15^, ^16^ Whether higher rates of acute COVID‐19 severity among PLHIV leads to a higher incidence of long‐COVID needs further studies. In the non‐COVID‐19 context, the data available are scant on the long‐term outcomes among PLHIV recovering from intensive care unit (ICU) stay.^17^ A meta‐analysis revealed significant short‐ and long‐term (≥90 days) mortality benefit following initiation/maintenance of ART in the ICU.^18^ In a case–control study of PLHIV admitted to ICU with sepsis (mostly pneumonia), mortality up to 1 year did not differ according to HIV status.^19^ Moreover, serial biomarker measurements indicating activation of cytokine network, vascular endothelium, and coagulation were similar in matched admission groups. However, none of the studies have reported the prevalence of post‐intensive care syndrome or other clinical symptoms after recovery from severe illness and hospital discharge.

Most individuals with moderate–severe COVID‐19 illness received short‐term steroids that were significantly associated with long‐COVID in our study in univariate analysis. While numerous adverse events are known with long‐term steroid use, a recent study from Taiwan revealed a higher incidence (up to 90 days) of GI bleed, sepsis, and heart failure after short‐term steroid use (<14 days).^20^ Among PLHIV on boosted protease inhibitors, even short‐term low‐dose steroids can lead to high plasma cortisol levels and may increase the risk of subsequent adverse events.^21^

Cough and fatigue were the major symptoms associated with long‐COVID in our study. Most studies among non‐HIV population have reported these to be the major long‐term symptoms.^10^, ^22^, ^23^, ^24^ Other symptoms in our study included shortness of breath, sleep disturbances, depression, and anxiety although at a lower frequency. Many of the symptoms associated with long‐COVID among PLHIV may overlap with the presentation of opportunistic infections/non‐AIDS conditions or antiretroviral toxicities. For example, NP symptoms like sleep disturbances, depression, and anxiety are common toxicities associated with efavirenz and INSTIs.^25^ Cognitive issues described as “brain fog” among long‐COVID may overlap with HIV‐associated neurocognitive disorders.^26^ It is essential for physicians to assess the temporal relationship and carefully evaluate these symptoms before attributing them to long‐COVID.

Our study has several limitations. Apart from being a single‐center study, information on symptoms relied on self‐report and subject to recall bias. We did not include control groups of HIV‐negative with COVID‐19 and HIV‐positive without COVID‐19. However, routine screening for HIV infection among all COVID‐19 individuals is not recommended in India. Information on the presence of symptoms before COVID‐19 diagnosis were not collected. Finally, formal tools for assessing depression and anxiety, laboratory and/or radiological evaluations were not routinely performed at screening.

In conclusion, we document a high prevalence of long‐COVID among PLHIV with moderate–severe acute COVID‐19 illness in western India. However, this rate is not higher than that historically reported among non‐HIV‐infected individuals. Integration of long‐COVID diagnosis and care in ART clinics needs to be established and further studies on understanding the pathophysiology and appropriate management are urgently needed.

## AUTHOR CONTRIBUTIONS

Sanjay Pujari designed the study and drafted the manuscript; Sunil Gaikwad recruited patients; Abhishek Chitalikar abstracted data from electronic records; Digamber Dabhade performed laboratory tests; Kedar Joshi and Vivek Bele performed statistical analysis. All authors commented on the draft manuscript and approved the final manuscript.

## Supporting information

Supporting information.

## Data Availability

The data that support the findings of this study are available from the corresponding author upon reasonable request.
