# Supplementary material for: Long‐coronavirus disease among people living with HIV in western India: An observational study
Source: Immun Inflamm Dis. 2021 Jun 2;9(3):1037–43. doi: 10.1002/iid3.467 (PMC8239760; doi:10.1002/iid3.467)
Supplement: Supplementary file 1 — Supporting information. [file IID3-9-1037-s001.docx]

POST-COVID SYMPTOM CHECKLIST

Name

ID number:

Date of assessment:

| SYMPTOMATIC |  | ASYMPTOMATIC |  |
| --- | --- | --- | --- |

**General**

| Fever | **Yes/No** |
| --- | --- |
| Chills | **Yes/No** |
| Body ache | **Yes/No** |
| Fatigue | **Yes/No** |
| Weakness | **Yes/No** |
| Myalgia | **Yes/No** |
| Joint pains | **Yes/No** |
| Sweats | **Yes/No** |
| Rash | **Yes/No** |

**Neurologic**

| Headache | **Yes/No** |
| --- | --- |
| Loss of smell | **Yes/No** |
| Loss of taste | **Yes/No** |
| Dizziness | **Yes/No** |
| Confusion | **Yes/No** |
| Sleep disturbance | **Yes/No** |
| Memory Loss | **Yes/No** |
| Anxiety | **Yes/No** |
| Depression | **Yes/No** |

**Respiratory**

| Cough Dry | **Yes/No** |
| --- | --- |
| Cough Productive | **Yes/No** |
| Sore throat | **Yes/No** |
| Sinus congestion | **Yes/No** |
| Shortness of breath (rest) | **Yes/No** |
| Shortness of breath (exertion) | **Yes/No** |
| Chest pain | **Yes/No** |
| Chest tightness | **Yes/No** |
| Wheezing | **Yes/No** |

**Gastrointestinal**

| Nausea | **Yes/No** |
| --- | --- |
| Anorexia | **Yes/No** |
| Vomiting | **Yes/No** |
| Diarrhea | **Yes/No** |
| Constipation | **Yes/No** |
| Abdominal pain | **Yes/No** |

Other symptoms (as reported by patient, not in the list above)
